# Maintenance Therapy in Acute Myeloid Leukemia: Current Perspectives and Future Directions

**DOI:** 10.3390/curroncol33060369

**Published:** 2026-06-18

**Authors:** Pilar Velarde, Asmaa Aloufi, David Sanford

**Affiliations:** Bone Marrow Transplant Program, Vancouver General Hospital, Vancouver, BC V5Z 1M9, Canada; pilar.velarde@bccancer.bc.ca (P.V.); asmaa.aloufi@bccancer.bc.ca (A.A.)

**Keywords:** acute myeloid leukemia, maintenance therapy, measurable residual disease, oral azacitidine, FLT3 inhibitors, allogeneic hematopoietic stem cell transplantation, relapse prevention, targeted therapy

## Abstract

The management of acute myeloid leukemia (AML) remains characterized by high relapse rates despite advances in induction and consolidation therapy. Relapse prevention represents a major unmet need, particularly in patients ineligible for allogeneic hematopoietic stem cell transplantation (allo-HSCT) or at high risk of post-transplant recurrence. This review examines current evidence supporting maintenance strategies following intensive chemotherapy or allo-HSCT, with emphasis on measurable residual disease (MRD)-guided approaches and targeted therapies. We summarize data from randomized and phase II/III trials evaluating hypomethylating agents, FLT3 inhibitors, IDH inhibitors, and immunotherapeutic strategies in post-remission settings. Oral azacitidine (CC-486) demonstrated overall survival benefit in older patients in first complete remission who were not transplant candidates, establishing a standard of care in this population. In FLT3-mutated AML, post-transplant maintenance with sorafenib and gilteritinib reduces relapse risk, with emerging evidence supporting MRD as a predictive biomarker for benefit. Other targeted agents and immunotherapies have shown promising early-phase results, although confirmatory data are limited. Ongoing phase III studies will clarify optimal patient selection, treatment duration, and integration with transplantation, aiming to transform post-remission management from passive surveillance to precision-based relapse prevention.

## Simple Summary

Acute myeloid leukemia is an aggressive blood cancer with a high risk of returning even after patients achieve remission with intensive treatment. Preventing relapse remains one of the biggest challenges, especially for individuals who are not candidates for stem cell transplantation or who remain at high risk after transplant. In recent years, maintenance therapy—lower-intensity treatment given after remission—has emerged as a promising strategy to reduce relapse risk. New targeted medicines and oral treatments have shown encouraging results in selected groups of patients. This review summarizes current evidence supporting maintenance approaches and discusses how risk-adapted strategies could improve long-term outcomes and direct future research and clinical practice in acute myeloid leukemia.

## 1. Introduction

Despite recent advances in the treatment of acute myeloid leukemia (AML), many patients relapse after initially achieving complete remission (CR) with intensive chemotherapy (IC) [1]. Patients at high risk of relapse, either due to high-risk genomic profiles or persistent measurable residual disease (MRD), are generally recommended to undergo allogeneic hematopoietic stem cell transplantation (allo-HSCT), which remains the only potentially curative option for most [1].

However, not all patients who achieve remission with front-line IC undergo allo-HSCT due to factors such as AML risk status, eligibility criteria, lack of access to treatment, toxicities experienced during IC, comorbidities, and patient preference. Even among those who undergo allo-HSCT in first CR, the relapse rate remains approximately 30–40% within the first 2–3 years [2].

In acute lymphoblastic leukemia (ALL), the role of maintenance therapy is well understood and has been established as a critical component of therapy. In AML, the concept of maintenance therapy was described over 50 years ago, but the more widespread use of maintenance in AML is more recent [1,2,3]. Maintenance and pre-emptive therapy are two main categories of interventions which can be used following IC or post- allo-HSCT, which aim to reduce relapse risk trying to balance against potential toxicity. Pre-emptive therapy is initiated in response to early molecular or immunologic evidence of relapse such as MRD positivity or declining donor chimerism, whereas maintenance therapy may be given to a broad group at higher risk of relapse, with the purpose to prolong remission and improve survival while allowing for acceptable quality of life. There is overlap between both strategies and although there is no formal definition of maintenance therapy, this is generally given over relatively longer periods of time and is consequently less intensive and requires a more tolerable side-effect profile. With both strategies, the risk of relapse must be balanced with potential for toxicity and concerns about overtreatment. For this review, we will focus on maintenance strategies, occasionally referring to pre-emptive therapy in situations where these strategies overlap [4].

**Figure 1:**
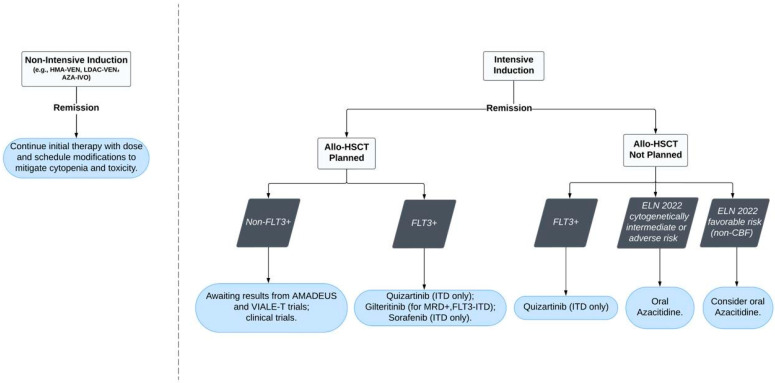
Recommendations for maintenance therapies in acute myeloid leukemia.

In AML, maintenance therapy is most frequently used and investigated in the following two main settings: the first includes patients who achieve remission after induction and consolidation chemotherapy but do not undergo allo-HSCT due to risk status, eligibility, preferences, lack of donor availability, or disease characteristics. The second setting is in patients following allo-HSCT, particularly those at high risk of relapse, such as those with adverse genetic profiles or with MRD positivity, with the goal of enhancing the graft-versus-leukemia effect (GVLE) and reducing post-transplant relapse [5,6,7]. The currently approved and/or guideline-recommended maintenance therapies are shown in Figure 1 and described in Table 1 and Table 2. However, there are still several questions about the use of maintenance such as the following: which AML patients may benefit most from maintenance and what are the optimal treatments as well as duration for this therapy?

## 2. MRD Guiding Maintenance

MRD represents the presence of residual leukemic cells below the threshold of morphological detection and is a powerful prognostic biomarker in AML. MRD positivity should not be interpreted as equivalent to morphologic relapse, but rather as the persistence or re-emergence of these cells below the threshold of conventional morphologic detection identifying a population at substantially increased risk of subsequent relapse. Detectable MRD after induction or consolidation strongly predicts relapse risk, whereas MRD negativity is associated with longer overall survival (OS) and relapse-free survival (RFS), making it a potentially valuable tool for guiding post-remission therapy [8,9]. While MRD positivity frequently precedes overt disease recurrence, not all patients with detectable MRD will experience immediate clinical relapse. Due to this strong correlation, MRD is being increasingly evaluated as a tool to individualize maintenance therapy decisions. An ideal MRD biomarker should be specific to leukemic cells, stable over the disease course, relevant for monitoring, and resistant to clonal heterogeneity or evolution. Advances in MRD detection—including multiparameter flow cytometry (MFC), quantitative polymerase chain reaction (qPCR), real-time qPCR (RT-qPCR), digital droplet PCR (ddPCR), and next-generation sequencing (NGS)—now allow detection of residual leukemic cells at sensitivities ranging from 10^−4^ to 10^−6^ [8,9].

The major challenge remains to identify the most sensitive and specific method for each AML subtype to guide maintenance therapy decisions effectively. According to the recent European Leukemia Network (ELN) 2025 guidelines, MRD monitoring should be performed broadly for a patient with AML following IC and before and after allo-HSCT. These guidelines include recommendations for molecular monitoring methodology for patients with mutated *NPM1*, *FLT3-ITD*, core-binding factor leukemia and *KMT2A* rearranged leukemia as well generally recommending MFC for other AML subsets. These recommendations highlight that MRD persistence can be used to identify high-risk patients who may benefit from maintenance or pre-emptive therapies, although the optimal intervention in most cases is still unknown.

Accumulating clinical evidence suggests that high-risk MRD-positive patients, particularly those harboring *FLT3-ITD* mutations, are the most likely to benefit from maintenance strategies following chemotherapy or transplantation. In contrast, the benefit of maintenance in MRD-negative patients remains less certain, although relapse rates of approximately 30% have still been reported in this group [9]. Ongoing and recently completed trials, such as MORPHO, help to clarify the predictive role of MRD in guiding targeted maintenance approaches and will be discussed later in the targeted therapy section.

For patients without actionable molecular targets, the role of MRD-guided maintenance is less clearly defined. In this context, pre-emptive treatment strategies have gained interest. The RELAZA2 trial demonstrated that initiating hypomethylating agents at the time of MRD positivity, rather than waiting for morphologic relapse, is feasible and may delay disease progression [10]. Similarly, the QUAZAR AML-001 trial provided important insights into the relationship between MRD dynamics and clinical outcomes. Patients receiving oral azacitidine (CC-486) showed higher rates of MRD clearance compared with placebo (37% vs. 19%) [11], and conversion to MRD negativity was associated with significantly longer OS (median OS 41.3 months vs. 9.0 months in patients with persistent MRD positivity; HR 0.21, 95% CI 0.14–0.32) [3]. Moreover, CC-486 appeared to be effective in prolonging remission duration regardless of initial MRD status [3,12].

Therefore, MRD currently serves more as a tool to guide pre-emptive intervention rather than as a definitive determinant for maintenance therapy selection [13]. However, as previously mentioned, in *FLT3-ITD*-positive AML, detectable MRD seems to identify a high-risk subgroup in which maintenance therapy may offer significant clinical benefit.

Further prospective studies are needed to define optimal MRD thresholds, timing of intervention, and the most effective therapeutic agents in this evolving treatment paradigm.

## 3. Hypomethylating Agents

Hypomethylating agents (HMAs) have been investigated as maintenance in AML in several clinical trials likely due to the broad applicability of these drugs and generally tolerable side-effect profile. CC-486 is currently the only non-targeted therapy approved by both the Food and Drug Administration (FDA), European Medicines Agency (EMAs) and Health Canada (HC) for maintenance therapy in AML patients who achieve CR or CRi following induction chemotherapy and are ineligible for allo-HSCT [3]. The QUAZAR AML-001 study leading to this approval included patients with AML in CR1, age 55 or older, intermediate or adverse cytogenetics who were not candidates for allo-HSCT [3]. Patients were randomized to CC-486 given at 300 mg per day for 14/28 days cycle versus placebo. The primary endpoint for the study was OS and this was 24.7 months in the CC-486 arm vs. 14.8 months in the placebo arm (*p* < 0.001). In longer-term follow-up of the study, 3-year OS was 37.4% in the CC-486 arm vs. 27.9% in the placebo arm. Factors associated with longer OS included: mutated *NPM1*, intermediate risk cytogenetics and MRD-negativity by flow cytometry (<0.1%) at the time of enrolment [14].

In the Dutch–Belgian Hemato-Oncology Cooperative Group (HOVON97), the effect of subcutaneous azacitidine was studied following IC; notably these patients did not receive any consolidation therapy before study entry [15]. In this open-label study, patients in CR/CRi were randomized to azacitidine 50 mg/m^2^ for 5/28 days for 12 cycles or observation. The study’s primary endpoint was disease-free survival (DFS) and this was significantly higher in the azacitidine arm (64% vs. 42% at 12 months, *p* = 0.04), although OS was not significantly different.

Subcutaneous azacitidine was also studied as maintenance in the post-allo-HSCT setting in a single-center clinical trial conducted by MD Anderson Cancer Center [16]. In the study, patients were randomized 1:1 to azacitidine given at 32 mg/m^2^ for 5/28 days or observation and there was no significant difference in RFS or OS between treatment arms. One recent randomized, controlled trial evaluated the effect of prophylactic recombinant human G-CSF (rhG-CSF) combined with minimal-dose decitabine on relapse in patients with high-risk, MRD-negative AML after allo-HSCT. In this open-label, multicenter phase II study, 204 patients who were MRD-negative post-transplant were randomized to receive either rhG-CSF plus low-dose decitabine maintenance or no intervention. The combined regimen significantly reduced the 2-year cumulative incidence of relapse (15.0% vs. 38.3% in controls; HR 0.32) and was associated with increases in lymphocyte subsets implicated in GVLE activity without increasing chronic graft versus host disease (cGVHD) [17].

Long-term hypomethylating agent plus venetoclax (HMA/VEN) therapy is not formally considered maintenance treatment, but in patients with sustained remission and MRD negativity it may serve a maintenance-like role. A subset of patients achieves prolonged remissions and OS exceeding five years, prompting interest in defining the optimal treatment duration. Retrospective data from 29 AML patients showed that over 50% of those who electively discontinued therapy remained in remission, with a median treatment-free remission of 45.8 months. Durable outcomes were most commonly observed in patients with MRD-negative status and favorable molecular features such as NPM1 or IDH2 mutation [18]. However, evidence supporting treatment discontinuation remains limited to small, retrospective, non-randomized studies, and prospective MRD-guided discontinuation trials ongoing before this approach can be adopted as standard practice.

## 4. FLT3 Inhibitors

FLT3 mutations are one of the most commonly found molecular aberrations in AML. *FLT3*-internal tandem duplications (*FLT3*-ITD) are present in approximately 25% of adults, and *FLT3*-tyrosine kinase domain (*FLT3*-TKD) mutations constitute 10% of AML cases [19,20]. Both *FLT3* mutations lead to constitutive activation of *FLT3* receptors. These mutations are associated with clinically proliferative disease and have historically been associated with higher relapse and worse OS; according to 2022 ELN guidelines patients with *FLT3*-ITD are now classified as intermediate risk regardless of *NPM1* mutation status or allelic ratio, unless adverse cytogenetic features are also present [21]. Nevertheless, even after allo-HSCT, risk of relapse remains relatively high and there is a need for improved therapeutic strategies in this population [20,22]. The first generation of FLT3 inhibitors are multi-targeted kinase inhibitors and include midostaurin, sorafenib, and lestaurtinib, which also demonstrated in vitro inhibition of the FLT3 receptor. Second-generation FLT3 inhibitors include quizartinib, crenolanib, and gilteritinib, which have more selective inhibitory activity, as well as higher potency, compared to first-generation compounds [22]. Gilteritinib and crenolanib are active for *FLT3*-ITD and TKD mutations whereas quizartinib is active only for *FLT3*-ITD [23].

Sorafenib is a multikinase inhibitor used in treatment of renal and hepatic carcinoma but has also been studied as maintenance therapy for AML, where it has shown a reduction in relapse and improvements in RFS and OS. In a phase I post-transplant study, 22 patients received sorafenib, with the maximum tolerated dose (MTD) determined to be 400 mg twice daily. In the phase II SORMAIN trial, 83 patients were randomized to sorafenib (*n* = 43) or placebo (*n* = 40) as post-transplant maintenance. Sorafenib was given at a target dose of 400 mg twice daily for a 2-year period, although a significant number of patients were given attenuated doses to mitigate side effects. Sorafenib significantly improved 2-year RFS compared with placebo (85% vs. 53.3%; HR 0.256, 95% CI 0.10–0.65; *p* = 0.002). Subset analysis suggested benefit in MRD-negative and MRD-positive patients, although this was based on small numbers of patients [24]. A randomized phase III trial from China (Xuan et al., *n* = 202) also supports sorafenib maintenance [25]. Patients in this study received either sorafenib 400 mg twice daily (*n* = 100) or placebo (*n* = 102) until day 180 post-transplant. With a median follow-up of 60 months, sorafenib was associated with improved OS (72.0% vs. 55.9%; HR 0.55, 95% CI 0.34–0.88; *p* = 0.011), improved leukemia-free survival (LFS) (70.0% vs. 49.0%; HR 0.47, 95% CI 0.30–0.73; *p* = 0.0007), and reduced relapse risk (15.0% vs. 36.3%; HR 0.33, 95% CI 0.18–0.60; *p* = 0.0003), without increasing non-relapse mortality (15.0% vs. 14.7%; HR 0.79, 95% CI 0.39–1.62; *p* = 0.98) or the incidence of GVHD [25]. Sorafenib appeared to have efficacy in both MRD-negative and -positive groups, although MRD positivity was associated with worse outcome overall in multivariate analysis. In both the SORMAIN and phase 3 studies, the sorafenib appeared to be tolerable for most patients but was associated with higher rates of study drug discontinuation and skin toxicity, which is a known toxicity of the drug [26,27]. In Canada, there is no Health Canada approval for sorafenib, and the drug is not reimbursed by provincial funding plans.

Gilteritinib maintenance has been supported through single-center and retrospective experiences included in meta-analyses and real-world data [28]. A post hoc analysis of the phase III ADMIRAL trial evaluating patients with *FLT3*-mutated relapsed/refractory AML initially reported benefit for gilteritinib maintenance after HSCT. This analysis found that patients who resumed gilteritinib after allo-HSCT had lower relapse rates and improved OS compared with those who did not, and the drug was generally well-tolerated. However, the analysis was limited by a small sample size and the lack of a second randomization at the maintenance phase [29]. The efficacy of gilteritinib as post-transplant maintenance was further evaluated in the randomized phase III MORPHO (BMT CTN 1506) trial [30]. This study enrolled 356 patients in CR1, more than half of whom had received FLT3 inhibitors with initial therapy pre-transplant. Gilteritinib was administered at 120 mg daily for up to 24 months, with RFS as the primary endpoint. For the overall cohort, gilteritinib was associated with a higher RFS, although the difference was not statistically significant (HR 0.679; 95% CI, 0.459–1.005; *p* = 0.0518). Importantly, 24 months after transplant, a subgroup of patients with detectable pre- or post-transplant MRD (~50% of patients), assessed using a highly sensitive PCR-NGS *FLT3*-ITD assay (LOD ~ 1 × 10^−6^), derived significant RFS benefit from gilteritinib (HR 0.515; 95% CI, 0.316–0.838; *p* = 0.0065). In contrast, MRD-negative patients achieved favorable outcomes without additional therapy, suggesting that MRD status can help identify patients who might safely avoid maintenance. Myelosuppression was the most common adverse event and the primary cause of early treatment discontinuation [30]. Overall, this identified MRD as a predictive biomarker for this treatment [24,25] and Clinical Practice Guidelines in Oncology (NCCN Guidelines^®^) [31] recommend gilteritinib as the preferred post-transplant maintenance option for AML with *FLT3*-ITD in CR1 without MRD negativity by ultrasensitive assay pre-transplant, and it is also recommended for *FLT3*-TKD mutations. Consensus document from the ELN-DAVID MRD Working Party also recommends gilteritinib maintenance for patients found to be MRD-positive (*FLT3*-ITD by UHS-NGS MRD) either before or around day 30 after allo-HSCT, particularly in those with a single *FLT3*-ITD clone and concurrent *NPM1* mutation, based on subgroup analysis of this trial (recommendation A15) [32].

A phase 2 study (GOSSAMER) also evaluated gilteritinib as maintenance therapy in patients with *FLT3*-ITD AML in CR1 following induction or consolidation therapy without plan for allo-HSCT [33]. Patients in this study were randomized 2:1 to receive gilteritinib or placebo given at a dose of 120 mg/day for up to 2 years. Although the primary endpoint of RFS was not significantly different between gilteritinib and placebo arms (HR 0.74; 95% CI, 0.41–1.34; *p* = 0.16), a trend toward potential benefit was observed, with RFS rates of 68.5% versus 55.3% at 1 year and 51.8% versus 44.9% at 2 years for gilteritinib versus placebo, respectively.

In the phase 3 RATIFY trial, patients in remission after consolidation continued maintenance with midostaurin or placebo according to their assigned treatment group. OS and event-free survival (EFS) were significantly improved with midostaurin plus standard chemotherapy compared with placebo, with similar rates of severe adverse events between groups. Four-year OS was 51.4% with midostaurin versus 44.3% with placebo [34]. A post hoc sensitivity analysis censoring for allo-HSCT showed a 24.3% lower risk of death with midostaurin, although the difference in four-year OS rates was not statistically significant (63.7% vs. 55.7%; *p* = 0.08). A post hoc landmark analysis including all patients who started maintenance, regardless of prior midostaurin exposure, showed no significant differences in OS or DFS between midostaurin and placebo during or after the 12 maintenance cycles. Median OS was not reached for either arm (HR: 0.80; 95% CI, 0.50–1.28; *p* = 0.3528), and median DFS was 39.4 and 56.3 months for midostaurin and placebo, respectively (HR: 0.99; 95% CI, 0.67–1.48; *p* = 0.9744). In the phase II RADIUS trial [35] *FLT3*-ITD-mutated AML patients receiving allo-HSCT were randomized to receive midostaurin for twelve 4-week cycles or standard-of-care treatments. The trial reported that midostaurin was well-tolerated and did not increase the risk of GVHD with gastrointestinal toxicities being the most common adverse events, consistent with its pre-transplant profile. The study enrolled only 60 patients and did not meet its primary endpoint and 18-month RFS was 89% in the midostaurin arm and 76% in the standard-of-care (SOC) arm (HR 0.46 [95% CI, 0.12–1.86]; *p* = 0.27) [35]. These findings have not led to formal recommendations for use, and the benefit of midostaurin maintenance remains uncertain.

Quizartinib, recently approved for frontline treatment of *FLT3*-ITD-mutated AML, has also been evaluated as maintenance therapy. In the QuANTUM-First study, quizartinib demonstrated an OS benefit compared with placebo [36]. Approvals were based on the phase III QuANTUM-First trial, which randomized 539 patients aged 18–75 years with newly diagnosed *FLT3*-ITD AML to quizartinib or placebo plus intensive 7+3 induction chemotherapy and High-Dose Cytarabine (HiDAC) consolidation (±allo-HSCT), followed by up to 3 years of quizartinib maintenance. Compared with placebo, quizartinib prolonged OS (31.9 vs. 15.1 months; HR 0.78; 95% CI, 0.62–0.98; *p* = 0.032) and showed a trend towards improved OS when censoring for allo-HSCT (HR 0.75; 95% CI, 0.56–1.01). CR duration was also longer with quizartinib versus placebo (median 38.6 vs. 12.4 months; HR 0.62; 95% CI, 0.45–0.86). Among the 89 patients who received quizartinib or placebo as maintenance after consolidation, the HR for OS was 0.4 (95% CI, 0.19–0.84). Rates of grade ≥ 3 adverse events were similar across arms (~90%), but grade ≥ 3 neutropenia (18% vs. 9%) and discontinuation due to adverse events (AEs) (20% vs. 9%) was increased in the experimental arm [36]. These findings support a potential benefit of quizartinib maintenance in patients who did not undergo allo-HSCT. Overall the positive results of the QuANTUM-First trial led to the FDA and EMA approval of quizartinib in 2023 for use in combination with cytarabine and anthracycline induction, cytarabine consolidation and as monotherapy maintenance in newly diagnosed *FLT3*-ITD AML [37,38]. In contrast, in a subset analysis of patients undergoing HSCT there was not clear benefit of quizartinib maintenance in this setting with no significant difference between groups. As the QuANTUM-First results were published after the last ELN update (2022), there are no formal ELN maintenance recommendations although quizartinib maintenance is included as an option following consolidation and allo-HSCT in NCCN guidelines [31] and in the provisional funding agreement (PFA) of the Canadian Drug Agency (CDA).

Additionally, crenolanib has been studied in a phase II trial in *FLT3-ITD* or *FLT3-TKD (D835)*-positive AML, assessing progression-free survival (PFS). The study included 30 patients; all enrolled patients underwent allo-HSCT and 20 of them received crenolanib as maintenance for at least 28 days. This study provides preliminary evidence that crenolanib post-allo HSCT is feasible and safe, but the lack of randomized controlled data prevents making definitive recommendations [39]. A randomized trial of crenolanib versus midostaurin plus chemotherapy in younger patients is ongoing and may provide more information.

## 5. Other Targeted Agents

IDH inhibitors have also been investigated as maintenance therapy. Ivosidenib and olutasidenib are oral inhibitors targeting mutant IDH1 and are approved by the FDA for R/R AML [40,41]. Ivosidenib is also approved in combination with azacitidine in the frontline setting for older and unfit patients with newly diagnosed AML by the FDA, Health Canada and in Europe [42,43]. Enasidenib is an oral inhibitor of mutant IDH2 approved by the FDA for R/R AML [44] and previously also by Health Canada but this was withdrawn following the results of a negative phase 3 study [44,45]. Both ivosidenib and enasidenib have been studied post-allo-HSCT. In a phase I trial, enasidenib was administered to 19 patients at 100 mg daily for 12 cycles starting 30 to 90 days post-transplant, achieving a two-year PFS of 69% and OS of 74%, with a cumulative incidence of relapse of 16% and limited GVHD events [46]. Similarly, ivosidenib at 500 mg daily was used post-allo-HSCT in 16 patients, resulting in a two-year PFS of 81%, OS of 88%, cumulative incidence of relapse of 19%, and non-relapse mortality of 0% [47]. Both drugs demonstrated promising safety profiles with limited toxicity and low risk of GVHD, supporting their consideration as maintenance therapy post-HSCT in *IDH1*- and *IDH2*-mutated AML, although larger prospective trials are required to confirm these results. A randomized phase II study of post-HSCT maintenance with ivosidenib vs. placebo in IDH1-mutant AML has been registered (NCT06707493) [48]. For non-transplant candidates, use of IDH inhibitors outside of clinical trials is not yet established. A phase 1 study of olutasidenib maintenance following HSCT is also currently recruiting (NCT06668584) [49].

Eprenetapopt, a first-in-class small-molecule p53 reactivator, has been tested in combination with azacitidine as maintenance therapy post-HSCT in patients with *TP53*-mutated AML or myelodysplastic syndrome (MDS) [50]. Phase II study (NCT03931291) [51] enrolling 55 patients, of whom 33 received maintenance treatment, administered eprenetapopt at 3.7 g once daily intravenously on days 1–4 combined with azacitidine 36 mg/m^2^ on days 1–5. The one-year RFS and OS probabilities were 60% and 79%, respectively, with median RFS and OS of 12.5 and 20.6 months [50]. The two parallel phase II trials (NCT03072043 and NCT03588078) [52] had also shown encouraging complete remission rates of 40–50% and molecular remission rates of 38% [53]. Treatment was well-tolerated with no increase in GVHD. These results were superior to previously published outcomes in *TP53*-mutated AML or MDS patients undergoing HSCT without subsequent maintenance, although the phase III trial (NCT03745716) [54] did not confirm efficacy.

Glasdegib, a Hedgehog pathway inhibitor, was used in a pilot study administering glasdegib for up to one year post-HSCT and demonstrated poor tolerability with frequent treatment interruptions and a relapse incidence of 55% at one year, leading investigators to conclude that it did not meaningfully reduce relapse risk [55]. Histone deacetylase inhibitors (HDACis) such as panobinostat have shown moderate antileukemic activity in advanced AML and MDS. In a phase I/II study of post-transplant panobinostat maintenance, 2-year OS and RFS were 81% and 75%, respectively, with toxicities largely reversible after drug interruption and no increased incidence of GVHD [56]. Vorinostat in combination with low-dose azacitidine is currently undergoing clinical evaluation (NCT03843528) [57].

Regarding maintenance failures, therapy with gemtuzumab ozogamicin (GO) was incorporated in the EORTC-GIMEMA AML 19 trial, in which low-dose GO was given monthly to elderly or unfit patients [58]. In this phase III trial, 237 patients received GO induction followed by maintenance GO if there was no progression of disease. Results improved median OS with GO compared to best supportive care, including transfusions and hydroxyurea [58]. Subsequent trials such as NOPHO AML 2004 [59] and HOVON 43 [60] did not show differences in DFS or OS, and GO is therefore not recommended for use as maintenance.

Tipifarnib was also explored as maintenance therapy based on the role of the RAS signaling pathway in myeloid malignancies. In a phase II maintenance trial, tipifarnib was given twice daily in patients with AML in CR1 and poor-risk cytogenetic or molecular features, showing a median DFS of 13.5 months [61]. The randomized phase III E2902 trial did not cross the pre-specified boundary to call the study positive, therefore failing to demonstrate improvement in DFS in the tipifarnib arm (8.9 vs. 5.3 months) [62], and it has not been FDA-approved [62].

## 6. Immunotherapies

In the setting of immunotherapy, multiple studies have explored prophylactic or pre-emptive strategies following IC or allo-HSCT. Although some of these studies may not be considered maintenance therapy in a strict sense, this approach is clinically important and warrants further investigation. Advances in MRD monitoring may further guide decision-making, potentially allowing these immunotherapeutic strategies to be tailored based on individual relapse risk. Recent studies investigating mechanisms of immune escape in AML after allo-HSCT have highlighted the distinctive immunologic microenvironment that develops post-transplant [63]. These findings suggest that future maintenance approaches in AML could harness immune system activity through monoclonal antibodies, bispecific antibodies, and other cellular therapies to suppress post-remission MRD [64].

Donor lymphocyte infusion (DLI) remains a well-established cellular immunotherapy used to elicit GVLE following allo-HSCT [65,66,67]. Prophylactic or preemptive DLI represents the most widely used immunotherapeutic strategy for relapse prevention after allo-HSCT [66,68]. Whether the benefit of DLI is greater with prophylactic compared with pre-emptive administration remains unclear. A recent comparative study demonstrated that prophylactic DLI administered early post-transplant (median 51 days) resulted in higher 2-year LFS (56.3% vs. 40.5%) and markedly reduced 2-year cumulative incidence of relapse (23.4% vs. 48.8%) compared to preemptive DLI triggered by MRD positivity [69]. These findings were corroborated by another multicenter study demonstrating higher 3-year PFS (63.4% vs. 53.0%) and significantly lower cumulative incidence of relapse (25.3% vs. 36.7%) with prophylactic versus preemptive DLI [70]. Nevertheless, both studies were limited by relatively small sample sizes and retrospective designs, and earlier matched-pair analyses suggested that the benefit of prophylactic DLI may be largely confined to patients with high-risk disease features. In this context, a registry-based matched-pair analysis from the European Society for Blood and Marrow Transplantation (EBMT) reported that while prophylactic DLI did not improve outcomes in the overall cohort, patients with high-risk AML experienced superior OS compared with matched controls (69.8% vs. 40.2%) [71]. Despite potential advantages, prophylactic DLI carries a significant risk of GVHD, particularly when given early [65]. EBMT registry study also reported cumulative incidences of grade 2–4 acute or cGVHD of 30.7% for prophylactic DLIs versus 11.9% for preemptive, with 6% of patients dying from DLI-induced GVHD [65]. Prophylactic DLI may be considered as early as day +90 to +100 post-transplant in patients without active GVHD or infection who have been off immunosuppression for at least one month; however, it does not reliably prevent early relapse and lacks a standardized dosing schedule [65].

Combining DLI with HMAs such as azacitidine has also shown promising results as a post-transplant maintenance or pre-emptive strategy, with acceptable tolerability and reductions in relapse rates in retrospective cohorts [72,73]. Current EBMT recommendations suggest considering prophylactic preemptive DLI for patients with high-risk biological features, refractory or advanced disease at transplant, or those receiving ex vivo T cell-depleted grafts, with the first one typically given after resolution of immunosuppression (>30 days), generally between 4 and 6 months post-allo-HSCT, and with dosing escalated every 4–6 weeks, guided by MRD, chimerism, and GVHD status [74].

Interferon alpha (IFNα) is a known antitumoral agent exerting both direct and indirect effects on leukemia cells. IFNα can reduce tumor cell proliferation by limiting the secretion of growth-promoting cytokines, increasing the immunogenicity of AML cells, modulating human leukocyte antigen (HLA) expression and activating cell-mediated cytotoxicity of T cells, NK cells, and dendritic cells towards tumor cells [75,76]. It has also been investigated in the post-allo-HSCT setting as a preemptive strategy rather than true maintenance. In a prospective pre-emptive-strategy study (NCT02027064) [77] of patients with t(8;21) AML and *RUNX1/RUNX1T1* MRD positivity after allo-HSCT, treatment was associated with relatively low GVHD rates and an MRD clearance rate of 21.5% at more than 3 months from initiation [78]. In non-transplant AML, IFNα did not demonstrate a survival advantage when used as maintenance therapy. Two initial randomized clinical trials, one from Finland by Palva [79], and the other from the UK (MRC AML11 trial), failed to show a beneficial effect of IFNα maintenance on DFS or OS. However, a more recent Chinese retrospective study of 84 patients with favorable-risk AML found that IFNα maintenance (not MRD-driven) for 12–18 months led to improved 4-year RFS of 87% versus 56% and OS of 94% versus 76% compared to a historical control cohort [80]. A phase 3 clinical trial (NCT06802718) is indeed testing IFNα in adults with favorable-risk AML and positive MRD, reflecting renewed interest in this approach for selected patient populations [81]. While early maintenance studies of IFNα in unselected AML populations were largely negative, emerging data suggest the possible role of preemptive or targeted strategy in biologically defined, favorable-risk or MRD-positive patients, claiming for further evaluation. A phase I-/II-pegylated IFNα trial (NCT02328755) in 36 high-risk AML patients undergoing allo-HSCT administered every 2 weeks starting day −1 of transplant regardless of MRD status suggested potential reduction in early relapse [82]. The incidence of relapse was 39% at 6 months, which was sustained through 1-year post HSCT. The incidence of transplant-related mortality was 13% and severe grade III-IV GVHD occurred in 11%. No randomized trial of pegylated IFNα is currently registered.

Interleukin-2 (IL-2) was one of the earliest immunomodulatory agents evaluated as a maintenance therapy in AML. IL-2 regulates T cell differentiation and NK cell proliferation, activating antileukemic T and NK cells to reduce relapse risk. However, multiple early randomized clinical trials and subsequent systematic reviews and meta-analyses of IL-2 monotherapy after IC (no-transplant candidates) found no evidence of extended DFS or OS compared to control groups, indicating IL-2 monotherapy did not provide meaningful survival benefit [83]. When combined with HDC (high-dose cytarabine), a multicenter randomized phase III trial of 320 adult AML patients in first complete remission after induction and consolidation therapy, not proceeding to allo-HSCT, evaluated maintenance with HDC plus low-dose IL-2 versus observation. Patients received subcutaneous injections of HDC and IL-2 twice daily in alternating cycles over 18 months, typically 3 weeks of treatment followed by 3–6 weeks of rest. In this trial, 3-year LFS was significantly higher in the HDC/IL-2 arm compared with the control arm (40 vs. 26%; *p* = 0.01), although there was no statistically significant difference in OS [84]. These results led to EMA approval in 2008 of the combination for maintenance in adult AML patients in first CR [85]. An initial negative recommendation by the EMA’s committee early in 2008 was subsequently reversed upon re-examination, and a positive opinion and full marketing authorization were granted later in 2008. The regimen is thought to enhance the immune system’s ability to eliminate residual leukemic cells by protecting IL-2-activated lymphocytes from oxidative suppression. Despite approval, IL-2/HDC maintenance therapy is not widely used in routine practice, largely because overall clinical benefit—especially in OS—was modest relative to toxicity concerns and the logistical burden of prolonged subcutaneous therapy.

Lenalidomide has been explored in multiple contexts. A single-center phase I dose-escalation study evaluated lenalidomide maintenance after allo-HSCT in high-risk MDS and AML patients using a “3 + 3” design starting at 5 mg daily, increasing in 5 mg increments up to 15 mg [86]. Lenalidomide was given for 21 days of a 28-day cycle for six cycles. Most common dose-limiting toxicities were lymphopenia, diarrhea, nausea, and neutropenia. Two patients developed acute GVHD (aGVHD) and five developed cGVHD. The maximum tolerated dose was 10 mg, with dose-limiting toxicities observed at 15 mg. Limitations included initiation at ~6 months post-transplant, by which time many high-risk patients may relapse. Overall, lenalidomide was well-tolerated with minimal GVHD and low relapse rates [86]. When combined with azacitidine in a phase Ib study, lenalidomide demonstrated reasonable tolerability and some efficacy, with median RFS of approximately 11–12 months and median OS ranging from 20 months to not reached [87]. The HOVON-SAKK-132 trial was a phase 3 clinical trial that investigated the addition of lenalidomide given following induction and consolidation chemotherapy as well as during maintenance therapy [88]. The study did not find any OS or EFS benefit in patients receiving lenalidomide and there was no apparent benefit for maintenance either [88].

Nivolumab, a PD-1 immune checkpoint inhibitor, has also been investigated. Post-allo-HSCT low-dose nivolumab (1 mg/kg) was explored in a small cohort but the study was prematurely discontinued due to high GVHD rates and toxicity [89]. In the randomized phase II REMAIN trial (NCT02275533), 80 patients with AML in first CR were not candidates for allo-HSTC and were enrolled for observation or nivolumab (3 mg/kg IV every 2 weeks for 46 doses)**.** The study did not demonstrate significant improvements PFS (13.2 months nivolumab vs. 10.9 months observation) or OS (53.9 months vs. 30.9 months, not statistically significant). There were substantially more adverse events on the nivolumab arm as follows: 27 patients (71%) had grade ≥ 3 AEs compared with five patients (12%) on observation (*p* < 0.001) [90]. Other immune checkpoint inhibitors have been explored in AML and post-transplant contexts. Ipilimumab induced responses in some post-transplant relapsed patients but was associated with severe immune-related adverse events, including fatal toxicity and significant GVHD, with similar increases in severe GVHD reported in lymphoid malignancies treated post-transplant. Sabatolimab, a TIM-3-targeting immunotherapy, has been evaluated post-HSCT in MRD-positive AML, with early data suggesting it is well-tolerated without inducing significant GVHD or immune toxicity. However, the STIMULUS-MDS1 trial did not meet its primary endpoints in higher-risk MDS. CR rates were similar between sabatolimab plus HMAs and placebo plus HMA (22% vs. 18%, *p* = 0.77), and there was no statistically significant improvement in PFS (11.1 vs. 8.5 months, HR 0.75, *p* = 0.10). The safety profile was manageable, with only one serious immune-mediated adverse event and one treatment-related death due to pneumonitis. Due to lack of efficacy demonstrated in clinical trials in AML and MDS, sabatolimab is no longer being developed in these indications [91].

WT1 (Wilms’ Tumor 1) is a highly attractive target for immunotherapy due to its strong expression in multiple malignancies, including AML. WT1 is expressed in 90–95% of AML blasts, in both peripheral blood and bone marrow, supporting the development of targeted therapies such as peptide vaccines [92,93]. In a phase II trial (NCT01266083) [94] of 22 patients in CR1 (median age 64), biweekly administration of a peptide vaccine—Galinpepimut-S (GPS), for six doses, with continuation to 12 doses in clinically stable patients—resulted in a 3-year OS of 47%, exceeding the prespecified endpoint of 34% and historical rates of 20–25% [95]. Median DFS was 16.9 months, and the vaccine was well-tolerated, with predominantly mild injection-site reactions. In a second trial (NCT00665002) [96] including 10 AML patients in second complete remission (CR2), GPS administration was associated with a median OS of 16.3 months versus 5.4 months in a historical cohort (*p* = 0.0175), along with a numerical improvement in median PFS (10.5 vs. 4.3 months). Durable responses were observed, with several patients remaining in remission throughout the treatment period. Overall, GPS was safe, immunogenic, and demonstrated encouraging survival outcomes, providing the rationale for further evaluation in phase III of REGAL trial (NCT04229979) [97].

Early-phase studies of engineered T-cell and NK-cell therapies for post-transplant relapse prevention in AML have demonstrated favorable safety and encouraging efficacy. Donor-derived TCR-engineered T cells targeting HA-1 (TSC-100) and HA-2 (TSC-101) were evaluated in phase 1 of ALLOHA trial (NCT05473910) [98], where 16 patients received one or two post-transplant infusions. No dose-limiting toxicities, cytokine release syndrome (CRS), ICANS, or TSC-related deaths were observed, and GVHD rates were comparable to controls [99]. At the July 2024 data cut, all TSC-treated patients remained MRD-negative and relapse-free, with complete donor chimerism, while 3 of 11 control patients relapsed. TSC therapy improved relapse-free probability at one year (*p* = 0.047) and EFS (HR 0.09, *p* = 0.025). Similarly, donor-derived or ex vivo-activated NK-cell therapies (e.g., KDS-1001; NCT05115630) were well-tolerated, supported engraftment, and showed low rates of aGVHD or infusion reactions. Overall, early results suggest these cellular immunotherapies may reduce relapse without increasing GVHD, but larger studies are needed to confirm long-term efficacy and optimal integration into post-transplant strategies.

## 7. Ongoing Clinical Trials

Several large, randomized phase III studies integrate targeted agents with intensive induction and consolidation, followed by maintenance, the studies are shown in Table 3. These include crenolanib versus midostaurin in newly diagnosed *FLT3*-mutated AML (NCT03258931) [100], with a sample size of 510 patients. This study aims to evaluate EFS over five years and allows transplantation when appropriate. The trial is active but no longer recruiting, and results are awaited given midostaurin’s role as the current standard of care. Similarly, gilteritinib versus midostaurin is being evaluated in the HOVON 156/AMLSG 28-18 trial (NCT04027309) [101], and ivosidenib and enasidenib versus placebo in *IDH*-mutated AML or MDS-EB2 is being studied in HOVON 150 (NCT03839771) [102]. Both trials have a primary endpoint of EFS and integrate intensive chemotherapy and transplant strategies, which may challenge midostaurin as the default partner to 7+3 depending on their outcomes. These three trials may also introduce a mutation-specific hierarchy within *FLT3*-mutated disease; however, maintenance decisions may still remain unclear, as no randomized trials specifically focused on maintenance strategies are currently registered.

Regarding the HOVON 150 trial, which enrolls nearly 1000 patients, results could establish targeted agent selection within intensive frontline therapy, not only in unfit or relapsed patients. The use of IDH inhibitors during frontline intensive chemotherapy would be a consideration if HOVON-150 demonstrates superior EFS for either ivosidenib or enasidenib. Because all three large induction trials allow allo-HSCT, their outcomes will inform whether certain targeted agents reduce relapse risk, potentially influencing which patients truly benefit from early allo-HSCT versus continued pharmacologic strategies.

In the post-allo HSCT transplant setting, targeted maintenance is being investigated in randomized and single-arm phase II trials using IDH inhibitors such as ivosidenib (NCT06707493) [48], enasidenib (NCT03728335; NCT04522895) [103,104], and olutasidenib (NCT06543381) [105], as well as the menin inhibitors revumenib (NCT06575296) [106] and ziftomenib (NCT06440135) [107]. The olutasidenib study aims to determine whether IDH1 inhibition post-transplant is feasible across AML, MDS, and chronic myelomonocytic leukemia (CMML). This is particularly relevant because *IDH* mutations are common, often persist at remission, and are biologically stable, making them attractive maintenance targets if long-term tolerability can be demonstrated.

All previously mentioned phase I/II post-HSCT trials may not immediately change clinical guidelines; however, they will add critical knowledge by defining whether targeted maintenance beyond FLT3 inhibition is tolerable and safe after allo-HSCT. These studies will help establish dosing windows, schedules, and toxicity profiles for menin inhibitors, IDH inhibitors, and antibody–drug conjugates during a vulnerable post-transplant period characterized by cytopenias, infections, GVHD, and drug–drug interactions. They may also help define optimal timing for initiation after HSCT—such as 30, 50, or 100 days post-transplant—by balancing immune reconstitution against relapse risk. Currently, timing decisions are largely based on expert opinion rather than prospective data, underscoring the need for evidence to standardize future protocols. Hopefully, consecutive phases of these trials would bring risk-adapted, mutation-directed maintenance strategies, transforming post-transplant care from passive surveillance into active, precision-based intervention.

There remains a substantial knowledge gap regarding the use of menin inhibitors in the post-allo-HSCT setting. A key unanswered question is whether these agents can suppress molecular relapse without impairing graft function. Serial MRD monitoring in small, genetically defined cohorts will be critical to determine whether post-HSCT targeted therapy can eradicate residual leukemic clones rather than merely delay overt relapse.

Several prospective studies investigating HMA-based maintenance strategies are also ongoing. The AMADEUS study (NCT04173533) [108] is a phase III placebo-controlled trial evaluating CC-486 maintenance for up to 12 months following allo-HSCT, with RFS as the primary endpoint. Enrollment was completed in April 2025, and results are pending. The VIALE-T study is a phase III open-label randomized controlled trial (NCT04161885) [109] comparing azacitidine plus venetoclax with best supportive care following transplant; enrollment was completed in September 2025, and results are anticipated.

Combination HMA-based strategies include venetoclax plus azacitidine in the VIALE-M trial (NCT04102020) [110]; oral decitabine/cedazuridine administered alone or in combination with venetoclax or targeted agents such as gilteritinib, enasidenib, or ivosidenib in mixed-intensity settings (NCT05010772) [111]; venetoclax plus cytarabine versus idarubicin (NCT04968015) [112]; and lenalidomide plus azacitidine in non-transplant candidates (NCT04490707) [113]. These trials aim to determine whether HMAs can function as long-term disease control strategies.

Mocravimod is an oral sphingosine-1-phosphate receptor modulator being investigated as post-transplant maintenance therapy to enhance GVLEs and reduce the risk of relapse following allo-HSCT. The phase III trial is evaluating its impact on RFS in patients with AML [114].

The ongoing REGAL trial compares GPS with investigator-selected best available therapy in patients with AML in second or later remission who are ineligible for allo-HSCT, with the aim of improving overall and relapse-free survival (NCT04229979) [97]. The study is overseen by an Independent Data Monitoring Committee; as of 26 December 2025, 72 events had been recorded, and final analysis will be performed after the 80th event. The results will provide further insight into the clinical activity of WT1-targeted vaccination strategies in AML.

In the post-transplant setting, immunomodulatory and adoptive cellular strategies are in early-phase development, including lenalidomide maintenance (NCT01254578) [115], ONC201 (NCT03932643) [116], γδ T-cell infusions (NCT05015426) [117], off-the-shelf natural killer cell products administered peri-HSCT (NCT05115630) [118], and T-cell receptor-engineered T-cell therapies delivered in the peri-transplant period (NCT05473910) [98]. Together, these studies will deepen understanding of immune surveillance and relapse biology in AML.

**Table 3:** Ongoing clinical trials, phase III.

Trial	Drug	HSCT	Short Description	Current Status (February 2026)
Randomized Phase III NCT03258931 [100]	Crenolanib vs. Midostaurin	IC ± allo-HSCT if appropriate	Population: adults aged 18–60 with confirmed FLT3-ITD and/or D835 mutations. Primary Outcome: EFS.	Active, not recruiting.
Randomized Phase III HOVON 156/AMLSG 28-18 NCT04027309 [101]	Gilteritinib vs. Midostaurin	IC ± allo-HSCT if appropriate	Population: adults (≥18 years) with newly diagnosed AML or MDS-EB2 with FLT3 mutation who are eligible for IC. Primary Outcome: OS.	Active, not recruiting.
Randomized Phase III HOVON150AML NCT03839771 [102]	Ivosidenib vs. Enasidenib	IC ± allo-HSCT if appropriate	Population: new AML or MDS-EB2 with a relevant IDH mutation. Primary Outcome: EFS.	Active, not recruiting.
Randomized Phase III NCT05429632 [114]	Mocravimod	Post-allo-HSCT	Population: adult patients (18–75 years) with AML undergoing allo-HSCT (includes patients in CR1 with high-risk or intermediate-risk disease and those in CR2). Primary outcome: RFS.	Active, recruiting.
Randomized Phase III REGAL NCT04229979 [97]	Galinpepimut	IC ineligible for allo-HSCT	Population: AML in second or later complete remission Primary Outcome: OS	Active, not recruiting.

IC, intensive chemotherapy; allo-HSCT, allogeneic hematopoietic stem cell transplantation; AML, acute myeloid leukemia; MDS-EB2, myelodysplastic syndrome with excess blasts-2; FLT3-ITD, FMS-like tyrosine kinase 3 internal tandem duplication; D835, FLT3 tyrosine kinase domain mutation at codon 835; IDH, isocitrate dehydrogenase; CR1, first complete remission; CR2, second complete remission; EFS, event-free survival; OS, overall survival; RFS, relapse-free survival.

## 8. Conclusions

Maintenance therapy in AML has evolved into an evidence-based treatment strategy that provides clinically meaningful benefit in selected patient populations. While reducing relapse risk without excessive toxicity remains the primary goal, optimal implementation continues to evolve.

From a practical standpoint, CC-486 should be considered standard of care in older patients with AML in CR1 who are not candidates for allo-HSCT, based on a demonstrated OS benefit in this setting. In *FLT3-mutated AML*, FLT3 inhibitors represent the most established maintenance approach, particularly in the post-transplant setting, where sorafenib and gilteritinib have shown clinically meaningful reductions in relapse risk, especially among MRD-positive patients.

In routine clinical practice, maintenance therapy is therefore best applied in a risk-adapted manner. Patients with high-risk disease features or persistent MRD positivity are the most likely to derive benefit and should be considered for maintenance strategies when supported by available evidence or clinical trial access. In contrast, MRD-negative patients without actionable mutations should generally be managed with observation, as there is currently insufficient evidence to support routine maintenance outside clinical trials.

The optimal duration and timing of therapy remain undefined. Post-transplant maintenance is commonly initiated between 30 and 100 days after allo-HSCT depending on immune reconstitution and relapse risk, while duration varies from fixed courses to continuation until progression or unacceptable toxicity. Importantly, interpretation of FLT3 inhibitor maintenance benefit is limited by trial designs in which targeted agents are introduced from induction onward, making it difficult to isolate the independent effect of the maintenance phase.

Emerging strategies, including IDH inhibitors, menin inhibitors, and immunotherapeutic approaches, are expanding the maintenance landscape but remain investigational. At present, these should be reserved for clinical trial settings. Integration of genomic profiling and serial MRD assessment will further refine patient selection and may allow treatment intensification or de-escalation based on individual relapse risk.

Overall, current evidence supports a precision-based, risk-adapted approach rather than uniform maintenance therapy. In practical terms, clinicians should prioritize:

(1) CC-486 in eligible non-transplant patients in CR1;
(2) FLT3 inhibitor maintenance post-transplant in FLT3-mutated disease, particularly if MRD-positive;
(3) Clinical trial enrollment for all other maintenance strategies;
(4) Observation for MRD-negative, lower-risk patients.

Ongoing phase III trials, including AMADEUS, VIALE-T, and multiple genetically targeted studies, will further define the role of maintenance therapy and may refine these recommendations. Cellular immunotherapies such as TCR-engineered T cells, NK cell products, and bispecific antibodies represent a promising future direction but remain experimental.

Finally, key unresolved questions include optimal treatment duration, timing relative to transplantation, sequencing with other targeted agents, and predictive biomarkers of both response and toxicity. Addressing these gaps will be essential to fully transition AML post-remission care toward a standardized precision-medicine framework.

### Limitations

This review has several limitations. Evidence supporting maintenance therapy in AML remains heterogeneous, with many studies being single-center, retrospective, or limited by small sample sizes. In addition, differences in patient populations, MRD methodologies, and transplant practices complicate cross-trial comparisons. As a result, the optimal selection of patients for maintenance therapy remains an area of active investigation.

## Figures and Tables

**Table 1:** Regulatory approval status and guideline recommendations for maintenance therapies in acute myeloid leukemia.

Agent	Regulatory Approval (Maintenance) EMA	Regulatory Approval (Maintenance) FDA	Regulatory Approval (Maintenance) HC	Clinical Guideline/Real-World Recommendations/CDA Reimbursement
Oral-AZA	YES	YES	YES	NCCN: treatment for adult patients (≥18 years) with non-CBF-AML who achieved remission with prior IC, completed no or some consolidation or a recommended course of consolidation, and are not planning to receive allo-HSCT (Category 1 recommendation for age ≥55 year); ELN: may be considered in patients unable to complete curative therapy; benefit post-consolidation or post-allo-HSCT remains unclear; CDA: reimburse for adult patients with AML in first CR/CRi following induction chemotherapy who are not candidates for intensive consolidation or allo-HSCT.
Midostaurin	YES	NO **	NO	NCCN: may be continued as maintenance if used during induction/consolidation (Category 2B); ELN: may continue as maintenance; value remains uncertain; Post-allo-HSCT: no formal recommendation; CDA: reimburse for newly diagnosed FLT3-mutated AML in combination with standard induction and consolidation chemotherapy, followed by single-agent maintenance for a defined duration (e.g., up to 12 months), consistent with clinical trial populations.
Quizartinib	YES	YES	YES	NCCN: Maintenance option following consolidation for FLT3-ITD AML (Category 2B). Recommended post-allo-HSCT; ELN or EBMT/ALWP: no recommendation post-allo-HSCT. CDA: reimburse with conditions for newly diagnosed FLT3-ITD AML in combination with standard chemotherapy where approved; maintenance use may be reimbursed only if aligned with the funded indication and clinical trial evidence.
Gilteritinib	NO	NO	NO	NCCN: post-allo-HSCT maintenance option for FLT3-mutated AML (Category 2B); ELN: no formal recommendation for post-allo-HSCT; CDA: Reimburse for relapsed/refractory FLT3-mutated AML (outside maintenance setting). No recommendation for reimbursement as maintenance therapy post-remission or post-allo-HSCT due to lack of supporting evidence.
Sorafenib	NO	NO	NO	NCCN: FLT3-ITD AML post-allo-HSCT maintenance (Category 2B; limited data/expert opinion); ELN 2022: no formal recommendation for post-transplant maintenance; EBMT/ALWP: recommended post-allo-HSCT for FLT3-ITD AML without active acute GvHD; CDA: no formal reimbursement recommendation for AML maintenance; use in this setting generally not funded, including post-allo-HSCT, due to limited high-quality evidence and lack of regulatory approval for this indication.

** FDA approval for Midostaurin covers use through induction, consolidation, and up to 12 months post-consolidation, but no strictly maintenance indication. AML, acute myeloid leukemia; allo-HSCT, allogeneic hematopoietic stem cell transplantation; CBF, core-binding factor; CR, complete remission; CRi, complete remission with incomplete hematologic recovery; IC, intensive chemotherapy; EMA, European Medicines Agency; EBMT, European Society for Blood and Marrow Transplantation; ALWP, Acute Leukemia Working Party; FDA, U.S. Food and Drug Administration; HC, Health Canada; CDA, Canada’s Drugs Agency, FLT3-ITD, FMS-like tyrosine kinase 3 internal tandem duplication; GvHD, graft-versus-host disease; NCCN, National Comprehensive Cancer Network.

**Table 2:** Approved/recommended therapies for maintenance in acute myeloid leukemia.

Trial/Phase	Pivotal Trial (PMID)	Patient Population	Setting	Timing/Duration of Maintenance	Main Efficacy Outcome
Oral Azacitidine (CC-486) Phase III	QUAZAR AML-001 (PMID: 33412009)	AML in CR/CRi after intensive chemotherapy, not proceeding to allo-HSCT	Post-consolidation	Within 4 months of CR/CRi after consolidation Until relapse/unacceptable toxicity (median 12 cycles)	Median OS 24.7 vs. 14.8 months; HR 0.69
Quizartinib Phase III	QuANTUM-First (PMID: 37116523)	Newly diagnosed FLT3-ITD AML after intensive chemotherapy ± allo-HSCT	Post-consolidation	After induction/consolidation Up to 36 cycles (3 years)	OS 31.9 vs. 15.1 months; HR 0.78
Sorafenib Phase II	SORMAIN (PMID: 32673171)	FLT3-ITD AML after allo-HSCT	Post-allo-HSCT	Started 60–100 days post-transplant Up to 24 months	2-year RFS 85% vs. 53.3%; HR 0.26
Sorafenib Phase III	Chinese multicenter randomized trial (PMID: 37414062)	FLT3-ITD AML after allo-HSCT	Post-allo-HSCT	Early post-transplant maintenance 6 months	5-year OS 72.0% vs. 55.9%; reduced relapse incidence
Gilteritinib Phase III	MORPHO/BMT CTN 1506 (PMID: 38471061)	FLT3-ITD AML in remission after allo-HSCT	Post-allo-HSCT	Initiated after hematologic recovery post-transplant Up to 24 months	Overall RFS benefit not statistically significant (HR 0.68; *p* = 0.052); significant benefit in MRD-positive patients (HR 0.52)

AML, acute myeloid leukemia; allo-HSCT, allogeneic hematopoietic stem cell transplantation; CR, complete remission; CRi, complete remission with incomplete hematologic recovery; FLT3-ITD, FMS-like tyrosine kinase 3 internal tandem duplication; HR, hazard ratio; IC, intensive chemotherapy; MRD, measurable residual disease; OS, overall survival; RFS, relapse-free survival.

## Data Availability

No new data were created or analyzed in this study. Data supporting the findings of this study are derived from publicly available sources, including the published literature and clinical trial registries such as PubMed and ClinicalTrials.gov, as well as guidelines from regulatory and professional organizations.

## Abbreviations

The following abbreviations are used in this manuscript:

ALWP	Acute Leukemia Working Party
AML	Acute Myeloid Leukemia
allo-HSCT	Allogeneic Hematopoietic Stem Cell Transplantation
CR	Complete Remission
CRi	Complete Remission with Incomplete Hematologic Recovery
DFS	Disease-Free Survival
EBMT	European Society for Blood and Marrow Transplantation
EFS	Event-Free Survival
ELN	European LeukemiaNet
EMAs	European Medicines Agency
FDA	U.S. Food and Drug Administration
FLT3	FMS-like Tyrosine Kinase 3
FLT3-ITD	FLT3 Internal Tandem Duplication
FLT3-TKD	FLT3 Tyrosine Kinase Domain
GVHD	Graft-versus-Host Disease
GVLE	Graft-versus-Leukemia Effect
HMA	Hypomethylating Agent
IC	Intensive Chemotherapy
IDH	Isocitrate Dehydrogenase
MRD	Measurable Residual Disease
NCCN	National Comprehensive Cancer Network
NPM1	Nucleophosmin 1
OS	Overall Survival
RFS	Relapse-Free Survival
